# 异基因造血干细胞移植治疗HIV相关Burkitt淋巴瘤1例报告并文献复习

**DOI:** 10.3760/cma.j.issn.0253-2727.2023.09.014

**Published:** 2023-09

**Authors:** 萌 王, 靖岚 张, 志磊 边, 伟杰 曹, 鼎铭 万, 尔烈 姜

**Affiliations:** 1 郑州大学第一附属医院血液科，郑州 450052 Department of Hematology, The First Affiliated Hospital of Zhengzhou University, Zhengzhou 450052, China; 2 中国医学科学院血液病医院（中国医学科学院血液学研究所），实验血液学国家重点实验室，国家血液系统疾病临床医学研究中心，细胞生态海河实验室，天津 300020 State Key Laboratory of Experimental Hematology, National Clinical Research Center for Blood Diseases, Haihe Laboratory of Cell Ecosystem, Institute of Hematology & Blood Diseases Hospital, Chinese Academy of Medical Sciences & Peking Union Medical College, Tianjin 300020, China

艾滋病是获得性免疫缺陷综合征（AIDS）的简称，人类免疫缺陷病毒（HIV）攻击人体免疫系统中最重要的CD4^+^ T细胞，可致人体不同程度的免疫功能缺陷，未经治疗的感染者在疾病晚期易合并各种严重感染，继发恶性肿瘤，最终全身衰竭而死亡[1],[2]。近年来进入联合抗反转录病毒治疗（combination antiretroviral therapy，cART）时代后，HIV感染者的预期寿命逐渐延长[3]，HIV相关淋巴瘤（HIV-related lymphomas, HRL）患者逐渐增多。由于HIV感染者免疫功能受到抑制，HIV对重建的供者T细胞的影响，预处理药物的毒性，cART药物和预处理药物及移植期间使用的其他药物之间的相互作用[4],[5]等，导致应用allo-HSCT治疗HRL较为少见。近期我们采用allo-HSCT成功治疗1例HIV相关Burkitt淋巴瘤（HIV-BL）患者，报道如下并进行文献复习。

## 病例资料

患者，男，41岁，因反复腹泻、腹痛于2021年9月就诊于当地医院，住院后检查发现HIV阳性，诊断为“获得性免疫缺陷综合征”，给予“TAF+3TC+DTG”抗HIV治疗。CT提示腹膜后腹主动脉及左侧髂动脉周围多发肿大淋巴结，行CT引导下腹部肿物穿刺活检术，病理：（腹膜后穿刺组织）免疫组化：BCL2（−），BCL6（弥漫+），CD10（弥漫+），CD21（−），CD3（T细胞+），CD30（−），CD5（T细胞+），CyclinD1（−），Ki67（>90％），MUM-1（部分+），P53（10％+），PAX-5（弥漫+），PD-L1（30％+），PD1（散在+），c-myc（90％+）。符合Burkitt淋巴瘤。PET-CT：横膈上下多发肿大淋巴结，腹膜后、髂血管旁淋巴结融合成团，部分层面与左侧腰大肌、髂腰肌分界欠清，范围11.6 cm×9.4 cm×24.6 cm（SUVmax 34.4）。病灶部分包绕左输尿管，继发左输尿管上段扩张。骨骼多发糖代谢增高，SUVmax 33.9，脾脏糖代谢弥漫略增高。综合考虑淋巴瘤Ⅳ期。血清EBV-DNA阴性；骨髓穿刺细胞学检查、流式细胞术、骨髓活检病理均未见异常淋巴细胞，融合基因及突变基因检查均为阴性。染色体核型46,XY（20）。于2021年9月行“利妥昔单抗（R）-hyper CVAD A”方案化疗，化疗后复查CT提示腹腔肿块较前缩小。2021年10月转至我院，11月行“R-Hyper CVAD B”方案化疗，后给予R-DAEL、R+hyper CVAD B、R+hyper CVAD A、R-Hyper CVAD B方案化疗4个疗程，期间多次检测高灵敏HIV-1 RNA（0.5～2）×10^2^拷贝。多次复查骨髓穿刺+活检均未见异常，先后6次行腰椎穿刺+鞘注化疗，脑脊液检查提示脑脊液蛋白含量增高，但多次行流式细胞术检查未见异常细胞。6个疗程化疗后复查PET-CT：膈上后群及腹膜后多发小淋巴代谢未见异常，左侧髂血管走行区小片状软组织影代谢稍活跃。于中华骨髓库找到无关供者（HLA 9/10相合，B位点不合）。2022年3月给予改良BU/CY+ATG方案预处理，移植期间继续cART治疗，2022年4月6日顺利输注供者单个核细胞（MNC）8.0×10^8^/kg，CD34^+^细胞5.5×10^6^/kg，移植后10 d中性粒细胞植入，12 d血小板植入，移植后12 d复查HIV-1 RNA未测出。移植后16 d患者出现Ⅲ度急性肠道GVHD，予以甲泼尼龙、CD25单抗等抗GVHD治疗，环孢素A改为静脉给药，患者腹痛、腹泻逐渐好转。随访至移植后10个月，多次复查高灵敏HIV-RNA未测出，移植后定期复查淋巴细胞亚群，移植后90 d复查CD8^+^T细胞及NK细胞恢复正常，CD4^+^ T细胞201/µl，B细胞持续为0。多次复查骨穿，骨髓检查未见异常，STR检测提示完全嵌合，复查PET-CT未见异常。

## 讨论及文献复习

与普通人群相比，HIV感染者（PLHIV）血液系统恶性肿瘤（包括非霍奇金淋巴瘤和霍奇金淋巴瘤）的发病率显著增高[1]–[2],[6]，自体造血干细胞移植越来越多地被用于治疗HIV相关血液病患者[7]。allo-HSCT是治疗高危恶性血液病的有效方法，但是考虑到AIDS患者的免疫缺陷与机会性感染的高风险等，目前关于allo-HSCT治疗HIV相关恶性血液肿瘤的报道较少。

Burkitt淋巴瘤具有高度侵袭性，起病时Ⅲ、Ⅳ期占85.2％，IPI评分高危、高中危组占74.1％[8]。HIV-BL是HRL的常见类型，文献报道，55.3％的患者有巨块病变，预后极差[9],[10]。本例患者为Burkitt淋巴瘤Ⅳ期，存在巨块病变，预后差，且给予cART治疗后，患者HIV复制稳定在较低水平，因此我们考虑给予allo-HSCT治疗。Arslan等[4]对多个数据库进行了全面的分析，纳入49例行allo-HSCT的HIV合并恶性血液病患者，移植后6、12个月总生存（OS）率分别为81.6％、56.6％，25例患者在随访中死亡。15例（60％）死于复发，3例（12％）死于感染，2例（8％）死于GVHD，5例（20％）死于其他原因，包括肾衰竭、急性呼吸系统疾病、肝衰竭。AIDS患者存在免疫缺陷，HIV持续复制感染供者细胞，可能会损害其淋巴细胞的正常功能，从而损伤移植物抗白血病（GVL）作用，因此可能导致AIDS患者移植后血液病复发风险增高。本例患者在移植后早期出现Ⅲ度急性肠道GVHD，与患者存在免疫缺陷及与非血缘供者B位点不合有关。

另外一项来自西班牙的多中心研究纳入22例行allo-HSCT的合并AIDS高危血液病患者，中性粒细胞、血小板植入率分别为91％、86％，中性粒细胞植入的中位时间为14.5（11～22）d，血小板的中位植入时间为20.5（6～480）d。其中两例患者表现出cART与移植药物的相互作用，1例表现为神经毒性和肝毒性，另1例表现为肾衰竭、横纹肌溶解症等。本例患者在移植后10 d中性粒细胞植入，移植后12 d血小板植入，与既往报道结果一致，提示多数AIDS患者可以耐受预处理方案毒性且造血重建无明显延迟。22例患者中19例（86.4％）在移植后0.2～77.5个月随访期中未检测到HIV复制。本例患者移植过程较为顺利，移植后12 d成功出仓，且HIV检测呈阴性。移植后停止cART时，HIV病毒载量增加，因此推荐在移植后仍应继续进行cART，注意cART药物与移植药物之间的相互作用。

CCR5是HIV入侵和感染人类细胞的受体，CCR5 Δ32/Δ32基因突变后，细胞缺乏CCR5受体，HIV就不能攻击CD4^+^ T细胞。Hutter等[11]报道了“柏林病人”，此患者2007年接受了CCR5Δ32/Δ32HLA匹配的非亲缘造血干细胞移植，AML得到有效控制，此后12年没有检测到HIV，但2020年9月死于白血病复发。此后一例感染HIV的霍奇金淋巴瘤患者（“伦敦病人”），接受了CCR5Δ32/Δ32造血干细胞移植，移植后长达30个月里没有检测到HIV[12]。以上研究提示，CCR5Δ32/Δ32造血干细胞移植可能在治愈血液系统肿瘤的同时逐渐清除HIV，实现AIDS的长期缓解。但是中国人中CCR5-Δ32突变非常少见，陈虎等[13]利用CRISPR基因编辑技术，在人成体造血干细胞上进行CCR5基因编辑，然后对一个患AIDS和急性淋巴细胞白血病（ALL）的27岁男性进行allo-HSCT，移植后获得形态学完全缓解，患者的T细胞呈现出对HIV的抵抗能力。

综上，本例患者的诊疗经过提示患有恶性血液病的AIDS患者进行allo-HSCT是可行的。allo-HSCT对HIV感染自然病程的影响尚需继续观察。
